# Reduced Risk of All-Cause, Cancer-, and Cardiovascular Disease-Related Mortality among Patients with Primary Malignant Cardiac Tumors Receiving Chemotherapy in the United States

**DOI:** 10.3390/curroncol30090618

**Published:** 2023-09-15

**Authors:** Duke Appiah, Carina R. Goodart, Grishma K. Kothari, Imo A. Ebong, Chike C. Nwabuo

**Affiliations:** 1 Department of Public Health, Texas Tech University Health Sciences Center, Lubbock, TX 79430, USA; 2 School of Medicine, Texas Tech University Health Sciences Center, Lubbock, TX 79430, USA; 3 Division of Cardiovascular Medicine, University of California, Davis, Sacramento, CA 95616, USA; 4 Department of Pathology, University of Colorado Anschutz Medical Campus, Aurora, CO 80045, USA; chike.nwabuo@cuanschutz.edu; 5 Ronin Institute, Montclair, NJ 07043, USA

**Keywords:** cardiac tumors, cardiovascular disease, chemotherapy, surgery, survival, mortality

## Abstract

Primary malignant cardiac tumors (PMCTs) are rare but lethal neoplasms. There are limited evidence-based treatment guidelines for PMCTs. We evaluated the relation of chemotherapy with mortality outcomes in patients with PMCTs in the United States. Data were from patients aged ≥ 20 years from the Surveillance, Epidemiology, and End Results program who were diagnosed with PMCTs from 2000 to 2020. Cox regression, competing risk, and propensity score analyses were performed to estimate hazard ratios (HR) and confidence intervals (CI). About 53% of the 563 patients with PMCTs received chemotherapy as the first course of treatment. During a mean follow-up of 24.7 months (median: 10), 458 deaths occurred with 81.7% and 9.4% due to cancer and cardiovascular disease (CVD), respectively. In models adjusted for sociodemographic and clinico-pathophysiological factors including histology, receipt of chemotherapy was associated with low risk for all-cause (HR: 0.56, 95%CI: 0.45–0.69), cancer (HR: 0.63, 95%CI: 0.50–0.80) and CVD mortality (HR: 0.27, 95%CI: 0.12–0.58). Patients who had both chemotherapy and surgery had the lowest risk for all-cause and cancer mortality. This study suggests that the subpopulations of patients with PMCTs who receive chemotherapy may have better prognosis than those who do not receive this therapy regardless of histology.

## 1. Introduction

Primary malignant cardiac tumors (PMCT) include sarcomas such as angiosarcomas, fibrosarcomas, synovial sarcomas, leiomyosarcomas, and rhabdomyosarcomas, as well as undifferentiated pleomorphic sarcomas, pericardial mesothelioma, and primary lymphomas [1,2]. Although rare, the incidence of PMCT has increased by more than 85% over the past four decades, owing to the increased availability and advancement of cardiac imaging instruments [3,4,5]. Comprising about 11% of all cardiac tumors [6], the incidence of PMCTs is reported to range from 34 to 131 per 10,000,000 persons [3,7].

Patients with PMCTs are usually asymptomatic in the early stages but often develop ventricular arrhythmias, heart failure, sudden cardiac death, and other cardiovascular diseases (CVD) within 48 to 80 months from diagnosis or identified at autopsy [3,8,9,10]. Accordingly, CVD is reported to be the leading cause of non-cancer deaths in patients with PMCTs [9,11]. Although PMCTs can present at any age, this lethal tumor is often diagnosed in the fifth decade of life, and has a poor prognosis compared to other cardiac tumors [3,10,12,13]. For instance, the 5-year survival of PMCTs is reported to be less than 18% across all histopathological types, with a median survival of 12 months [3,11,14,15,16,17].

There are currently limited evidence-based guidelines on the management of PMCTs due to limited information from large samples of patients enrolled in clinical or population-based studies [4]. Current treatment modalities are often palliative involving adjuvant chemoradiotherapy and radiation as well as complete surgical resection to reduce the risk of recurrence [2]. Adjuvant chemotherapeutic agents such as paclitaxel, doxorubicin, prednisone, cyclophosphamide, or vincristine when administered early in the management of PMCTs and used either as an auxiliary to surgical resection or in palliation of unresectable disease has been reported to reduce tumor size [2].

In order to gain a large sample of patients with PMCTs, most studies evaluating the impact of surgery and cancer therapeutics on long-term outcomes in this population often evaluate patients diagnosed across several decades. Thus, the limited number of studies that reported the benefits of adjuvant chemotherapy on survival among patients with PMCTs often included a small proportion of patients who were diagnosed with the disease in the modern era of cancer therapeutics [9,16,18]. An unintended consequence of studying patients with rare cancers over several decades is the potential for large heterogeneity in treatment regimens influencing the findings [19]. Furthermore, most prior studies did not adequately consider the potential influence of confounding factors related to treatment selection bias [9,14,16,17,18,20]. Also, there is very limited knowledge on the role of chemotherapy on cardiovascular outcomes among patients with PMCTs.

With chemotherapeutic agents constantly evolving in recent years [21], coupled with expanded indications for their use among cancer patients [22,23], it is important to understand the potential prognostic effects of chemotherapy on survival among patients with PMCTs. Therefore, we evaluated the relation of chemotherapy with all-cause, cancer-, and CVD-related mortality in a modern population-based cohort of patients with PMCTs in the United States.

## 2. Materials and Methods

### 2.1. Study Population

Data for this study were obtained from the Surveillance, Epidemiology, and End Results (SEER) program which covers approximately 34.6% of the United States population, and almost all of the incident cancers in its population-based cancer registries areas of Alaska, California, Connecticut, Georgia, Hawaii, Iowa, Kentucky, Louisiana, New Mexico, New Jersey, Utah, and Washington [24].

**Figure 1:**
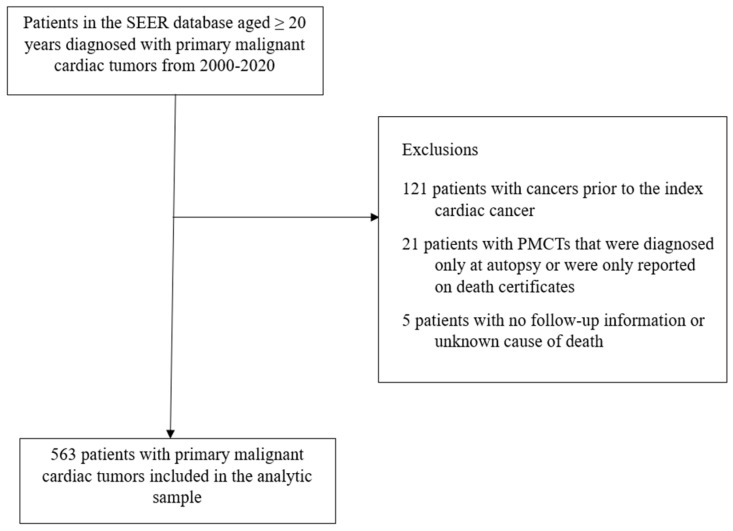
Patient selection with exclusion criteria, SEER 2000–2020.

The SEER registry uses the International Classification of Diseases for Oncology, 3rd Edition (ICD-O-3) codes for the diagnosis of cancer. The inclusion criteria for the current analysis were having being diagnosed with PMCTs in the modern treatment era, that is often defined as any time since 2000 and being diagnosed at the age of 20 or more years. Of the 710 participants diagnosed with malignant cardiac tumors based on SEER site code C38.0 (heart) from 2000 to 2020 and who met this inclusion criteria, the following exclusions were made: 121 patients with cancers prior to the index cardiac cancers as such cases could be secondary malignancies, 21 patients with PMCTs that were only diagnosed at autopsy or were only reported on death certificates, and 5 patients with no follow-up information or unknown cause of death. This resulted in an analytic sample of 563 patients (Figure 1). Since SEER is a de-identified publicly available database, institutional review board approval was not required for the present study.

### 2.2. Definition of Study Variables

The main exposure of interest in the current study was the first course of chemotherapy which was classified as receiving or not known to have received this treatment. In a validation study using linked SEER-Medicare data, the sensitivity, specificity, positive predictive value, and negative predictive value of the SEER database correctly identifying individuals who received chemotherapy within 4 months of diagnosis with cancer was 72%, 97%, 87%, and 92%, respectively [25].

The outcomes for the present study are all-cause, cancer-, and CVD-related mortality. Causes of death were defined based on International Classification of Diseases, Tenth Revision (ICD-10) codes. Cancer mortality was defined as with ICD-10 codes: C00-C97). CVD mortality was defined by any of the following causes of death (ICD-10 codes): diseases of heart (I00–I09, I11, I13, I20–I51), hypertensive heart disease (I10–115), cerebrovascular diseases (I60–I69), atherosclerosis (I70), aortic aneurysm and dissection (I71), and other diseases of arteries, arterioles, and capillaries (I72–I78). In SEER, the use of ICD codes to identify cause of death has been reported to have good validity [26].

The SEER database has the following information available: age at diagnosis reported per 5-year age groups, year of cancer diagnosis, sex, race and ethnicity, location (rural or urban) of patient’s residence, marital status, average annual median household income of patient’s county of residence, SEER summary stage of disease, histopathology, mode of diagnostic confirmation, cancer therapy, cause of death, and survival time. Geographic region was determined based on the location of cancer registry. Histopathology of PMCTs was classified as sarcoma, lymphoma, mesothelioma, and others with the latter two groups combined due to small samples. Average annual median household income was determined based on estimates from the American Community Survey for the year that the patient was diagnosed.

### 2.3. Statistical Analysis

Characteristics of participants at the time of cancer diagnosis according to receipt of chemotherapy were described using chi-square and Fishers exact tests. To estimate if mortality due to CVD among patients with PMCTs is higher than the expected estimate in the general population, age-standardized mortality ratios were calculated using the U.S. population during the period of 2000–2020 by dividing the observed and expected number of CVD events. In estimating cumulative incidence in time-to-event analyses, Kaplan–Meier product limit estimator and the log-rank test was used for all-cause mortality, whereas competing risk analysis was used for cancer- and CVD- related mortality with Gray’s test used to comparisons among patients who received chemotherapy to those who did not receive chemotherapy. Cox proportional hazards models and cause-specific hazard models were used to estimate hazard ratios for the association of chemotherapy with all-cause as well as cancer and CVD-mortality. Models were adjusted for age, year of diagnosis, sex, race and ethnicity, marital status, geographic region, median household income, tumor stage, histology, surgery, and radiation therapy. For all outcomes, interaction tests were performed for chemotherapy and surgery, chemotherapy and radiation, and chemotherapy and tumor histology, and reported whenever found to be statistically significant.

Several sociodemographic and clinico-pathophysiological conditions are known to influence the decision to initiate chemotherapy [27,28,29,30]. To control for such confounding factors and minimize bias related to the receipt of chemotherapy influencing the observed outcome, propensity scores were estimated by computing the probability of patients receiving or not chemotherapy using a logistic regression model. Variables included in the logistic regression model were age, year of diagnosis, sex, race and ethnicity, marital status, geographic region, median household income, tumor stage, histology, surgery, and radiation therapy. To match participants (1:1. match) who received chemotherapy to those who did not receive chemotherapy, the nearest-neighbor greedy matching algorithm with calipers set at 0.25 of the standard deviation of the logit of the propensity score was used. Patients were required to be matched exactly on tumor stage.

Balance in covariates before and after matching or after weighting by propensity scores using inverse probability of treatment was determined using an absolute standardized difference of <0.2 to represent negligible differences in the prevalence or mean distribution of covariates between matched pairs [31]. All previously estimated hazards ratios were recalculated by conducting propensity score matching and inverse probability of treatment weighting analyses, with the weights stabilized to prevent unreliable results due to the influence of extreme weights [32]. For all analyses, statistical significance was determined based on a two-tailed type 1 error of less than 0.05. Analyses were performed using the SEER*Stat version 8.4.1.2 software (Information Management Systems, Rockville, MD, USA), the SAS software version 9.4 (SAS Institute, Inc., Cary, NC, USA) and the R software (version 4.3.0; R Foundation for Statistical Computing) [33,34,35,36].

## 3. Results

The proportion of patients diagnosed with PMCTs increased over the 20-year period from 19.2% during 2000–2004 to 32.0% for 2015–2020. More than a third of patients with PMCTs were diagnosed at ages 45–64 years, with majority (60.4%) of the cases living in the West region of the United States at the time of diagnosis. The majority of tumors were sarcomas (70.2%) followed by lymphomas (27.3%) and mesothelioma together with other histologies (2.3%). The racial and ethnic distribution of patients were as follows: non-Hispanic White (58.4%), non-Hispanic Black (10.0%), Hispanic (19.2%), Asian or Pacific Islander (11.4%), and other race/unknown (1.1%). About 53% of patients received chemotherapy as the first course of treatment. Characteristics of patients according to receipt of chemotherapy are presented in Table 1. A greater proportion of patients who received chemotherapy were younger, married, had cancer stages described as distant at the time of diagnosis, had lymphomas, and did not receive radiotherapy. There was no difference in the proportion of patients who had surgery for the two groups.

**Figure 2:**
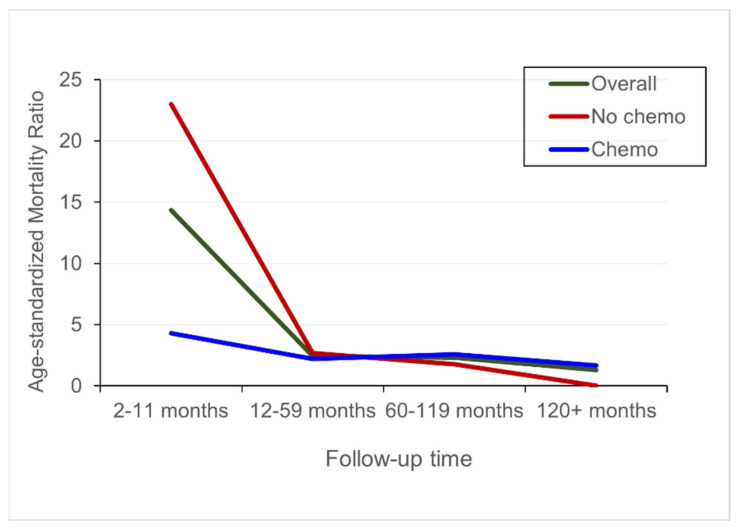
Standardized mortality ratios comparing CVD mortality for patients with primary malignant cardiac tumors to adults in the U.S. general population according to time since diagnosis and receipt of chemotherapy, 2000—2020.

**Figure 3:**
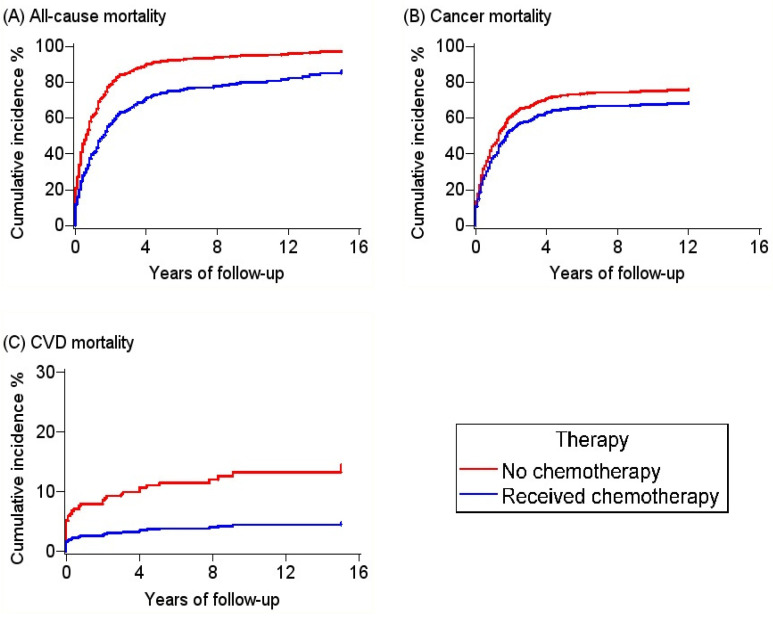
Cumulative incidence curves for all-cause, cancer-, and cardiovascular disease-related deaths among patients with primary malignant cardiac tumors, SEER 2000–2020. The *p* values for Gray’s test for equality of cumulative incidence functions were <0.01.

During a mean follow-up of 24.7 months (median: 10 months), 458 deaths (81.4%) were observed with 81.7% due to cancer (PMCTs) and 9.4% due to CVD. The age-standardized mortality ratio for CVD was 5.22. Thus, patients with PMCTs were 5.22 times statistically significantly more likely to die of CVD compared to adults in the general population. The excess CVD mortality among patients with PMCTs was highest within the first year after cancer diagnosis. However, the mortality ratio was lower for patients who received chemotherapy compared to those who did not receive chemotherapy (Figure 2). In time-to-event analysis, all-cause, cancer-, and CVD related mortality were lower among patients who received chemotherapy compared patients who did not receive chemotherapy (Figure 3).

The median (interquartile range) overall survival time for patients who received chemotherapy was 18.0 (16.0–21.0) months compared to 3.0 (2.0–5.0) months for patients who did not receive chemotherapy. In multivariable adjusted models (Table 2), compared to patients who did not receive chemotherapy, those who received chemotherapy had a 44% reduced risk for all-cause mortality (HR: 0.56, 95%CI: 0.45–0.69), 37% reduced risk for cancer-related mortality (HR: 0.63, 95%CI: 0.50–0.80), and 73% reduced risk for CVD mortality (HR: 0.27, 95%CI: 0.12–0.58).

**Figure 4:**
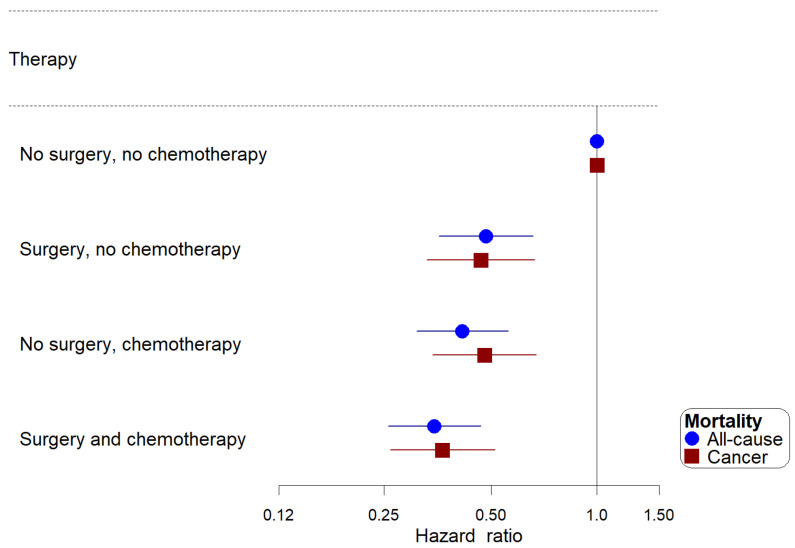
The independent and joint association of receipt of chemotherapy and surgery with all-cause and cancer mortality among patients with primary malignant cardiac tumors, SEER 2000–2020.

There was a significant interaction between chemotherapy and surgery with patients who had both treatments having the lowest risk for all-cause (HR: 0.35, 95%CI: 0.26–0.47) and cancer-related mortality (HR: 0.36, 95%CI: 0.26–0.51) (Figure 4). No interactions between chemotherapy and radiation or chemotherapy and tumor histology were identified for all outcomes.

**Figure 5:**
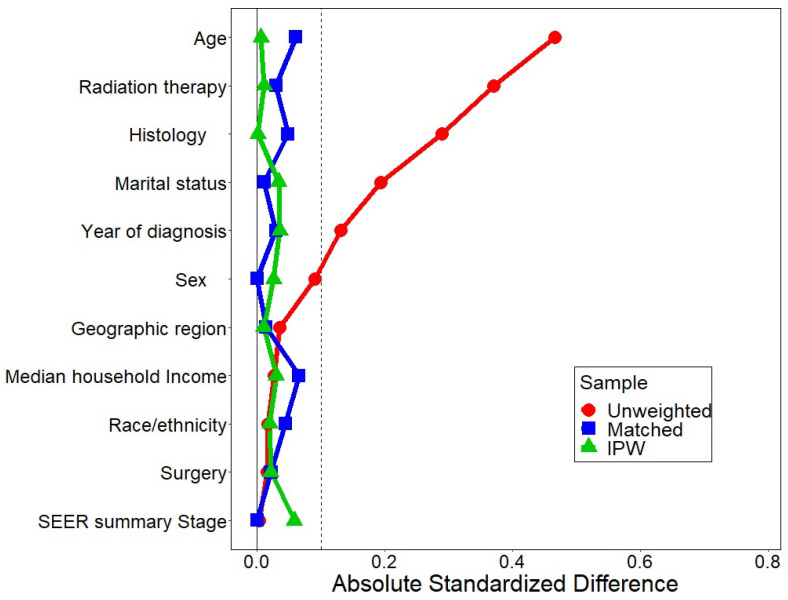
Absolute standardized differences for baseline covariates comparing treated to untreated patients in the original and the matched sample or after weighting for propensity scores using inverse probability of treatment weights. IPW: inverse probability of treatment weighting.

After propensity score matching, 185 patients who received chemotherapy were successfully matched to 185 patients who did not receive chemotherapy. A good balance of baseline characteristics was reached between the matched group with absolute values of standardized differences ranging from 0.0 to 0.142 (Table 3, Figure 5). The hazard ratios for the association of chemotherapy with mortality outcomes from propensity score matched and the inverse probability of treatment weighting analyses were similar to those obtained from models without propensity score adjustments, with the differences falling within ≤0.09 hazard ratios.

## 4. Discussion

In this 20-year population-based study of patients with PMCTs, it was seen that the subpopulation of patients who received chemotherapy as the first course of treatment, regardless of histology of PMCTs, had better prognosis as indicated by the lower risk of all-cause, cancer-, and CVD related mortality compared to patients who did not receive this therapy during the period of observation.

The results of the current study which used a modern cohort of patients with PMCTs agree with previous studies from SEER as well as other cancer registries that evaluated the role of chemotherapy on survival of patients with PMCTs [9,14,16,17,20]. For instance, the study by Bui et al., which had the same median follow-up of 10 months, like the current study, also reported the same (44%) reduction in all-cause mortality due to chemotherapy use among Black and White patients with PMCT in the United States, regardless of race [16]. Similarly, in developing nomograms to predict survival among patients with PMCTs, Guan et al. reported that chemotherapy was an independent prognostic factor for survival resulting in 41% and 37% reduced risk for all-cause and cancer-specific mortality [9]. Also, Yin et al., using SEER data, reported that after a median follow-up of 7 months, chemotherapy was independently associated with a 30% reduced risk of all-cause mortality among patients with PMCTs [17]. Unlike the present study, all these investigations included a relatively smaller sample of patients diagnosed with PMCTs in the modern era of cancer therapeutics where the chemotherapeutic agents used tend to be different to those used about 40 years ago [21,22,23]. There is scarcity of data in the modern era on the potential impact of chemotherapy on outcomes in patients with PMCTs. Among the few studies of patients with PMCTs diagnosed in the modern era, results from both the SEER database [20] and the National Cancer Database [14] all showed improved survival among patients with PMCTs who received chemotherapy.

Currently, there are limited standardized treatment guidelines for PMCTs [4]. To inform evidence-based recommendations, there is a great need for studies that extensively evaluate the role of cancer therapeutics on long-term outcomes in large cohorts of patients with this rare but lethal cancer. Surgery is often considered as the primary treatment of choice when tumors are detected early, and complete tumor resection leads to improvement in survival [14,37]. However, most patients present late with PMCTs which makes complete tumor resection either not possible or have variable success [14,38,39,40,41,42,43]. Chemotherapy is usually indicated for advanced cases of some PMCTs, or as a neoadjuvant therapy before surgery [20,38]. Among patients with primary cardiac lymphoma, chemotherapy is often the preferred primary treatment choice due to the sensitivity of lymphoma to chemotherapy [44]. Results from the present study and other recent studies suggest a benefit of chemotherapy on overall survival for patients with primary cardiac sarcoma [9,45,46]. For instance, in the current study, the median survival of patients with PMCTs who used chemotherapy was 18 months, compared to 3 months for patients who did not use this therapy. Similarly, a single site study of 31 patients with primary cardiac sarcoma reported that patients who received up to six regimens of chemotherapy had longer median survival (14.0 month) compared to patients who did not receive chemotherapy (2.4 months) [45].

Multimodality treatment involving surgery, radiation therapy, and chemotherapy has been previously reported to significantly prolong survival in patients with PMCTs [18,41,46]. In the current study, the median overall survival time among patients who received both chemotherapy and surgery was 20 months compared to 8 months for patients who had surgery alone. Sultan et al. reported that patients with Stage III PMCTs who underwent surgery with adjuvant chemotherapy and/or radiation had median survival of approximately 15 months compared to 3 months for patients with had surgery alone [14]. In the current study, however, we did not find any significant interaction between chemotherapy and radiotherapy for all evaluated outcomes, probably due to less than a quarter of the sampled patients receiving both chemotherapy and radiation therapy. The combination of chemotherapy with surgery resulted in significantly lower risk for all-cause and cancer-related mortality than having these treatments alone or no treatment. This evidence is supported by the observation that chemotherapy enhances surgical resection of tumors, and it is well tolerated among patients with PMCTs, even for right-side cardiac sarcomas that tend to be infiltrative and difficult to treat [46]. However, in a single-center study of 15 patients diagnosed with non-metastatic primary cardiac sarcomas from 1979 to 1995, although overall survival was significantly longer for patients with completely resected tumors (22 vs. 7 months), post-operative chemotherapy was not found to significantly improve overall survival [47].

It is widely known that the decision to initiate chemotherapy is based on factors such as age, race, comorbidities, tumor stage, grade, and patient/provider preferences [27,28,29,30]. These factors tend to make baseline characteristics of patients who receive chemotherapy differ systematically from those do not receive such treatment [32]. However, none of these aforementioned studies of patients with PMCTs implemented measures like propensity scores which reduces the presence of confounding related to the receipt of chemotherapy and allow for observational studies to mimics some peculiar characteristics of randomized controlled trials [31,32]. In the current study, methods that are known to have superior control of confounding over regression adjustments such as propensity score matching and inverse probability of treatment weighting analyses were implemented. Although there was a slight attenuation of the hazard ratios in these analyses, chemotherapy was still found to be inversely associated with all-cause, cancer-, and CVD-related mortality.

The findings of chemotherapy being associated with reduced risk of CVD mortality is novel, and significant since CVD is the leading cause of non-cancer deaths in patients with PMCTs [9,11]. In the present study, we observed that patients with PMCTs had a higher mortality due to CVD than the general population. These findings may seem counter intuitive since for more than four decades, conventional chemotherapeutics such as anthracyclines have been recognized to be cardiotoxic, resulting in myocardial mass loss that leads to progressive cardiac remodeling and dysfunction, and eventually heart failure [48]. In contrast to anthracyclines, other chemotherapeutics like trastuzumab have a high potential of reversibility of cardiac dysfunction that occurs during cancer treatment, and also results in low risk of progressive CVD even after several years of follow-up [48]. Nevertheless, the full spectrum of cardiotoxicity often becomes evident several months or years after the initial cancer treatment [48,49], Therefore, with the SEER database not reporting the various chemotherapy regimens received by patients, coupled with most patients with PMCTs not living for long after diagnosis, further studies evaluating the role of specific chemotherapeutics and CVD outcomes in patients with PMCTs are warranted. Such investigations, especially among patients who live more than a year after diagnosis of PMCTs, will enhance our understanding of the unique roles that the various chemotherapeutic agents play on survival in this patient population.

The strength of our study includes the use of a sample of patients with PMCTs diagnosed within the modern era of cancer therapeutics, who were sampled from the large population-based SEER database, a known reliable source of epidemiologic information on cancer in the United States. The following limitations of our study should be considered. First, the SEER database does not include information on specific chemotherapy regimens, duration of treatment, or dosage. Second, since SEER does not capture the use of treatment administered outside of the hospital setting [50], it is possible that some patient classified as not having received chemotherapy or unknown chemotherapy status in SEER may actually have received this therapy. Although a 10% rate of under-ascertainment of adjuvant chemotherapy is reported among patients with resected stage III colorectal cancer in SEER [50], a validation study of treatment assignments among a larger population of patients found high specificity and high negative predictive value for the classification of chemotherapy status in SEER [25]. If present in the current study, such under-ascertainment bias would have been expected to result in weak or null findings for the association of chemotherapy with all-cause, cancer-, and CVD related mortality. However, this was not the case. Third, propensity scores were used to achieve excellent balance of baseline characteristics between patients with PMCTs according to chemotherapy status. However, due to their unavailability in the SEER database, we could not account for the influence of other factors also known to influence the decision to receive chemotherapy. These include comorbidities at the time of cancer diagnosis, risk factors for cancer or CVD, patient preferences, and physician recommendations [27,28,29,30]. Of note, more than two-thirds of patients with PMCTs from the National Cancer Database were reported to have a Charlson comorbidity score of zero [14]. Finally, for rare and lethal cancers like PMCTs, the potential for misclassification of deaths, especially non-cancer deaths, cannot be entirely ruled out despite the SEER database reported to have good validity in the classification of causes of death [26].

## 5. Conclusions

The findings of this large study of patients with PMCTs suggest that chemotherapy has a positive impact on the prognosis of these rare and lethal cancers as it relates to all-cause, cancer-, and CVD-related mortality. These findings need validation from well-conducted randomized control trials that have the potential to balance patients on measured and unmeasured potential confounding factors related to the receipt of chemotherapy.

## Figures and Tables

**Table 1:** Characteristics of participants with primary malignant cardiac tumors according to receipt of chemotherapy, SEER registry (*n* = 563).

Characteristics	Chemotherapy		*p* Value
	No (*n* = 263)	Yes (*n* = 300)	
Age, years			<0.001
20–44	57 (21.7)	104 (34.7)	
45–64	89 (33.8)	122 (40.7)	
≥65	117 (44.5)	74 (24.7)	
Sex			0.353
Male	136 (51.7)	167 (55.7)	
Female	127 (48.3)	133 (44.3)	
Year of diagnosis			0.336
2000–2004	59 (22.4)	49 (16.3)	
2005–2009	57 (21.7)	70 (23.3)	
2010–2014	67 (25.5)	81 (27.0)	
2015–2020	80 (30.4)	100 (33.3)	
Race and ethnicity			0.565
Non-Hispanic White	155 (58.9)	174 (58.0)	
Non-Hispanic Black	22 (8.4)	34 (11.3)	
Hispanic	56 (21.3)	52 (17.3)	
Other	30 (11.4)	40 (13.3)	
Region			0.967
Midwest	13 (4.9)	16 (5.3)	
Northeast	43 (16.3)	53 (17.7)	
South	47 (17.9)	51 (17.0)	
West	160 (60.8)	180 (60.0)	
Marital status, married	127 (48.3)	173 (57.7)	0.026
Median household income			0.794
<$75,000	140 (53.2)	163 (54.3)	
≥$75,000	123 (46.8)	137 (45.7)	
Location, rural	22 (8.4)	33 (11.0)	0.293
SEER summary stage			<0.001
Localized	86 (32.7)	92 (30.7)	
Regional	71 (27.0)	79 (26.3)	
Distant	74 (28.1)	119 (39.7)	
Unknown/unstaged	32 (12.2)	10 (3.3)	
Histology			<0.001
Sarcoma	203 (77.2)	192 (64.0)	
Lymphoma	52 (19.8)	103 (34.3)	
Mesothelioma and others	8 (3.0)	5 (1.7)	
Surgery			0.822
Yes	129 (49.0)	150 (50.0)	
No	134 (51.0)	150 (50.0)	
Radiation therapy			<0.001
Yes	26 (10.0)	70 (23.6)	
No	235 (90.0)	227 (76.4)	

**Table 2:** Hazard ratios and 95% confidence intervals for the association of receipt of chemotherapy with all-cause, cancer-, and CVD-related mortality, SEER 2000–2020.

	All-Cause Mortality	Cancer Mortality	CVD Mortality
	HR (95%CI)	HR (95%CI)	HR (95%CI)
Before PS matching—unadjusted	0.52 (0.43–0.62)	0.59 (0.48–0.72)	0.23 (0.12–0.46)
Before PS matching—adjusted	0.56 (0.45–0.69)	0.63 (0.50–0.80)	0.27 (0.12–0.58)
After PS matching	0.65 (0.52–0.81)	0.71 (0.56–0.89)	0.30 (0.13–0.69)
IPTW	0.63 (0.52–0.76)	0.67 (0.55–0.82)	0.36 (0.19–0.68)

CI: Confidence interval, HR: Hazard ratio, IPTW: Inverse probability of treatment weight, PS: Propensity scores.

**Table 3:** Characteristics of participants with primary malignant cardiac tumors according to receipt of chemotherapy after propensity score matching, SEER registry (*n* = 370).

Characteristics	Chemotherapy		ASD	*p* Value
	No (*n* = 185)	Yes (*n* = 185)		
Age, years			0.056	0.894
20–44	53 (28.6)	57 (30.8)		
45–64	67 (36.2)	66 (35.7)		
≥65	65 (35.1)	62 (33.5)		
Sex			0.033	0.835
Male	98 (53.0)	101 (54.6)		
Female	87 (47.0)	84 (45.4)		
Year of diagnosis			0.025	0.901
2000–2004	33 (17.8)	34 (18.4)		
2005–2009	40 (21.6)	44 (23.8)		
2010–2014	52 (28.1)	46 (24.9)		
2015–2020	60 (32.4)	61 (33.0)		
Race and ethnicity			0.044	0.161
Non-Hispanic White	112 (60.5)	108 (58.4)		
Non-Hispanic Black	17 (9.2)	23 (12.4)		
Hispanic	13 (7.0)	24 (13.0)		
Other	40 (21.6)	28 (15.1)		
Region	3 (1.6)	2 (1.1)	0.037	0.929
Midwest	11 (5.9)	9 (4.9)		
Northeast	32 (17.3)	36 (19.5)		
South	33 (17.8)	33 (17.8)		
West	109 (58.9)	107 (57.8)		
Marital status, married	91 (49.2)	98 (53.0)	0.076	0.533
Median household income			0.000	1.000
<$75,000	101 (54.6)	101 (54.6)		
≥$75,000	84 (45.4)	84 (45.4)		
Location, rural	15 (8.1)	23 (12.4)	0.142	0.230
SEER summary stage			0.000	1.000
Localized	60 (32.4)	60 (32.4)		
Regional	55 (29.7)	55 (29.7)		
Distant	63 (34.1)	63 (34.1)		
Unknown/unstaged	7 (3.8)	7 (3.8)		
Histology			0.012	0.910
Sarcoma	129 (69.7)	128 (69.2)		
Lymphoma	48 (25.9)	56 (30.3)		
Mesothelioma and others	8 (4.3)	1 (0.5)		
Surgery			0.000	1.000
Yes	91 (49.2)	91 (49.2)		
No	94 (50.8)	94 (50.8)		
Radiation therapy, yes			0.000	1.000
Yes	159 (85.9)	159 (85.9)		
No	26 (14.1)	26 (14.1)		

ASD: Absolute standardized difference, SEER: Surveillance, Epidemiology, and End Results.

## Data Availability

Data used for this study are publicly available from the National Cancer Institute at https://seer.cancer.gov/ (accessed on 17 July 2023).
